# Dynamic changes in human THP-1-derived M1-to-M2 macrophage polarization during *Thelazia callipaeda* MIF induction

**DOI:** 10.3389/fimmu.2022.1078880

**Published:** 2023-01-11

**Authors:** Changzhu Yin, Juan Cai, Yanting Gou, Di Li, Hongri Tang, Lingjun Wang, Hui Liu, Bo Luo

**Affiliations:** Department of Parasitology, Zunyi Medical University, Zunyi, China

**Keywords:** *Thelazia callipaeda*, MIF, signal pathway, macrophage polarization, immune evasion mechanism

## Abstract

Macrophages are innate immune cells with essential roles in the immune response during helminth infection. Particularly, the direction of macrophage polarization could contribute to pathogen trapping and killing as well as tissue repair and the resolution of type 2 inflammation. This study establishes that the recombinant protein of *Thelazia callipaeda* macrophage migration inhibitory factor (*T.cp*-MIF) induces THP-1-derived macrophages to undergo M1 to M2 type dynamic polarization, using the methods of flow cytometry, real-time quantitative PCR, differential transcriptomic analysis and western blot. Interestingly, there was an increase in protein and mRNA expression of M1-type proteins and cytokines after the use of PI3K inhibitors, suggesting that the polarization state tends to favor the M1 type after M2 type inhibition. In conclusion, the dynamic polarization mechanism of *T.cp*-MIF-induced human THP-1-derived macrophages from M1 to M2 type is related to the binding of TLR4. It can first affect the M1 type polarization of macrophages by activating its downstream NF-κB pathway. Activation of the PI3K/Akt pathway and inhibition of NF-κB phosphorylation affects the M2 type polarization of macrophages.

## 1 Introduction

Human *Thelaziasis* is an arthropod-borne ocular disease, caused by *Thelazia callipaeda*, which is parasitic in the lacrimal ducts and conjunctival sacs and causes acute or chronic inflammation such as conjunctivitis and endophthalmitis (1). During chronic inflammation, the parasite’s ability to survive and proliferate in the host for long periods is closely linked to the complex immune evasion mechanisms that result from inflammation. Much progress in understanding parasite immunity and immune evasion mechanisms has been made. However, there is a limited understanding of immunity and immune evasion mechanisms in the interaction between ocular parasites such as *T. callipaeda* and its hosts. In addition, this parasite has many genes that are highly homologous to those of the host, which can replicate proteins derived from worms that help the parasite evade host immunity. MIF is a regulator with multiple immune effects, which can regulate the transcription of target genes through the activation of downstream molecules by phosphorylation and participate in a series of pathophysiological processes such as cell growth and migration and inflammation and immune response regulation. There is a lot of evidence that parasite-derived MIF is similar in structure and function to human-derived MIF, affecting macrophage polarization and immune evasion (2, 3).

Macrophages, as effector cells for antigen presentation and anti-infection, can be differentiated into classically activated macrophages (M1) and alternatively activated macrophages (M2) under different stimulation conditions. In parasitic infections such as *Neospora caninum* and *Clonorchis sinensis*, macrophages show predominant M2 activation or dynamic M1 to M2 polarization. Macrophages play a crucial role in parasite immune evasion by suppressing the inflammatory response, promoting chronic infection, and facilitating long-term parasitism (4–6). Macrophage polarization is regulated by many signaling molecules and pathways. Studies have shown that a variety of TLR ligands bind to TLRs and activate signaling pathways related to macrophage polarization, producing pro-inflammatory or anti-inflammatory factors, which in turn affect macrophage polarization (7, 8). The related signaling pathways of macrophage polarization mediated by TLRs mainly include NF-κB, PI3K/Akt, JAK/STAT6, p38/MAPK. Among them, NF-κB, p38/MAPK, and other pathways are involved in M1-type polarization (9, 10), while PI3K/Akt, JAK/STAT6, B7H3/STAT3, mTOR, and other pathways are involved in M2-type polarization (11–13).

*T.cp*-MIF inhibits THP-1 macrophage migration and induces the M2 phenotype polarization of THP-1 macrophages through TLR4 binding and PI3K-Akt pathway activation (14). Recombinant *T.cp*-MIF protein induces THP-1-derived macrophages to undergo M1 to M2 type polarization. Further, we want to know whether this change is a dynamic process. The critical time nodes of macrophage M1 to M2 transition were analyzed and the expression levels of some important molecules were verified. Based on the transcriptome data, we analyzed the related signaling pathways and found that this dynamic polarization was mediated by TLR4/NF-κB and TLR4/PI3K/Akt signaling pathways. This study therefore aimed to understand how parasite- derived MIF regulates and affects the process and direction of host macrophages polarization. We sought to provided some reference information to understand the immune regulatory mechanisms involved in *Thelazia callipaeda’s* interaction with its hosts.

## 2 Materials and methods

### 2.1 Expression and purification of *T.cp*-MIF protein

The expression and purification process of *T.cp*-MIF was described by Cai J (14). Genome-wide information on *T.cp*-MIF was obtained from (Gene ID Cluster-6025.14069). The *T.cp*-MIF gene is 620 bp in length—with an open reading frame of 345 bp, encoding 115 amino acids—and a molecular weight of about 14.37 kDa. The *T.cp*-MIF gene was inserted into the prokaryotic expression vector pET-SUMO (Novagen, Madison, WI, USA) to construct a pET-SUMO-MIF plasmid, which was expressed in *E.coli* BL21 (DE3) with 1 mmol/L isopropyl-β-D-thiogalactopyranoside (IPTG), and purified by HisLink™ affinity chromatography columns at a preservation concentration of 10 mg/mL.

### 2.2 Cell culture and induction

Human leukemia THP-1 monocytes were purchased from the Shanghai Cell Bank of the Chinese Academy of Sciences. THP-1 cells were cultured as suspension cells in RPMI1640 medium containing 10% fetal bovine serum, 1% double antibody, and 0.05 mM sodium pyruvate in a 37 °C, 5% CO_2_ cell culture incubator. One percent of the dual antibodies are composed of a penicillin-streptomycin solution. THP-1 cells in logarithmic growth phase cells in good growth condition were inoculated uniformly at a density of 1×106 cells/mL in a culture dish of 10 cm in diameter and incubated with PMA (100 ng/mL) (Sigma Corporation, USA) in the medium for 48 h to induce adherent macrophages and whether the induction was successful was determined by changes in cell morphology and surface markers. On this basis, 50 ng/mL *T.cp*-MIF was used for different times according to the experimental grouping.

### 2.3 Transcriptome sequencing and bioinformatic analysis

The induced THP-1-derived macrophages were cultured in complete medium (RPMI 1640 medium containing 10% fetal bovine serum, 1% double antibody, 0.05 mM sodium pyruvate) containing *T.cp*-MIF (50 ng/mL) for 24 and 48 hours, and THP-1-derived macrophages without *T.cp*-MIF were used as a control group. The experimental groups consisted of cells treated with *T.cp*-MIF for 24 and 48 hours, with 3 samples in each group. The steps of transcriptome sequencing library building included total RNA extraction, RNA quality detection and fragmentation, cDNA generation by reverse transcription, end complementation, addition A- tail, connector addition, PCR amplification, and Illumina machine sequencing. After sequencing, DESeq software was used for standardized processing. According to the criteria of difference multiple >2 and difference significance<0.05, differentially expressed protein-coding genes were screened for GO functional analysis and KEGG pathway enrichment.

### 2.4 Flow cytometry analysis

To detect the effect of *T.cp*-MIF recombinant protein on THP-1 macrophage polarization, THP-1 macrophages were collected and treated with fluorescein isothiocyanate (FITC)-labeled anti-human CD11b antibody, phycoerythrin (PE)-labeled anti-human CD14, phycoerythrin (PE)-labeled anti-human CD86 antibody and allophycocyanin (APC)-labeled anti-human CD206 were incubated together (Invitrogen, USA). The concentration of antibodies used in the flow cytometry analysis (of 100 5-µL tests in total) was 0.5µg/test. We added 50 ng/mL *T.cp*-MIF for induction, centrifugated the samples at 1200 rpm after 12 h, 24 h, 36 h, and 48 h, centrifuged for 10 min, discarded the supernatant, and added 200 µL of PBS to resuspend. The cells were incubated at 4 °C for 30 minutes, 1.5 mL of PBS was added and centrifuged at 1200 rpm for 10 minutes; the supernatant was discarded, and 300 µL of PBS were added to resuspend and mix the cells, which were transferred to a flow cytometer. The percentage of cells double labeled with CD11b and CD14 antibodies, which could be identified as macrophages, reached over 85%, and subsequent experiments could be carried out on this basis. If the percentage of CD11b and CD86 double positivity was above 85%, the population could be identified as M1 type macrophages, and if the percentage of CD11b and CD206 double positivity was above 85%, it could be identified as M2 type macrophages.

### 2.5 Real-time fluorescent quantitative polymerase chain reaction (RT-qPCR) analysis of the cytokine transcripts

Total RNA was isolated from macrophages in different groups using Trizol (Takara Corporation, Japan) and converted to cDNA. The reverse transcription system was prepared in a de-enzymatic octet tube and placed in the PCR instrument for reverse transcription. The reverse transcription system is shown in **Table 1**. Then, cDNA was obtained using PrimeScriptTM RT Reagent Kit according to the manufacturer’s instructions (Takara Corporation, Japan). Quantitative real-time PCR was conducted using a SYBR Premix Ex Taq Kit (Takara Corporation, Japan) and a quantitative fluorescence PCR instrument (Bio-Rad Corporation, USA). Fold changes in gene expression were calculated using the 2^−ΔΔCt^ method to calculate the relative mRNA expression of TNF-α and Arg1. The primer pairs used for these analyses are shown in **Table 2**. The PCR reaction system and procedures are shown in **Table 3**.

**Table 1:** Reverse transcription system (20 µL).

Component	Volume
5× PrimeScript Buffer	4 µL
PrimeScript RT Enzyme Mix I	1 µL
Oligo dT Primer (50 µM)	1 µL
Random 6-mers (100 µM)	1 µL
Total RNA	Depending on concentration

**Table 2:** Real-time qPCR amplification primers.

Gene	Primers
TNF-α	F:TTTTGCCAAGGAGTGCTAAAGA
	R:AACCCTCTGCACCCAGTTTTC
Arg1	F:GTGGAAACTTGCATGGACAAC
	R:AATCCTGGCACATCGGGAATC
GAPDH	F:GGAGCGAGATCCCTCCAAAAT
	R:GGCTGTTGTCATACTTCTCATGG

**Table 3:** RT-qPCR reaction system (25 µL).

Component	Volume
TB Green Premix Ex Taq II (2×)	12.5 µL
Forward Primer (10 µM)	1 µL
Reverse Primer (10 µM)	1 µL
Template (cDNA)	2 µL
RNase-Free ddH2O	Up to 25 µL

### 2.6 Western blotting

Cells of different groups were fully lysed on ice for 30 min using the ready-made RNAiso Plus lysis solution, gently scraped off with a clean cell scraper, and centrifuged for 15 minutes at 13,000 rpm at 4 °C. The supernatant was aspirated to obtain the cellular proteins, which were denatured by boiling and used for experiments. After the protein concentration was detected by the protein BCA method, the protein sample was added to the sample well to start electrophoresis. The upper layer of separated gum is 10% and the lower layer of concentrated gum is 4%. Protein samples were added to the sample wells (minimum quality assurance of 20 µg of protein per well). The electrophoresis was started after adding the appropriate amount of electrophoresis solution in the electrophoresis tank with an initial potential difference of 80 V. When the bromophenol blue ran into the separation gel (after about 40 min), the voltage was adjusted to 100 V until the end of electrophoresis. After electrophoresis, the gel was removed, and the excess gel was cut off according to the molecular weight of the protein. The gel was then placed, as per standard procedures, along with sponge pads, filter paper, and a PVDF membrane, into the transfer tank, so that the bands on the gel could be transferred to the PVDF membrane. The appropriate amount of pre-cooled transfer solution was added, and transferring was carried out at 300 mA for 90 min. The PVDF membranes were placed on a shaker for blocking at room temperature for 2 h at 45 rpm. The 5% blocking solution is composed of 2.5 g of skimmed milk powder and 50 ml of TBST.

After blocking, the corresponding primary antibodies such as TLR4 (Wuhan Sanying Biotechnology Co., Ltd.), NF-κB (Wuhan Sanying Biotechnology Co., Ltd.), p-NF-κB (Cell signaling technology, USA), PI3K (Wuhan Sanying Biotechnology Co., Ltd.), p-PI3K (Abcam, UK), Akt (Wuhan Sanying Biotechnology Co. Ltd.), and β-actin (Hangzhou Huaan Biotechnology Co., Ltd.) was separately added to the box (antibody concentrations were diluted according to the instructions), incubated at 4°C for over 12 h. After incubation, the membrane was washed 3 times with TBST buffer for 10 min each time. According to the antibody dilution ratio, the goat anti-rabbit (mouse) secondary antibody (Wuhan Sanying Biotechnology Co., Ltd.) was prepared with TBST buffer and incubated at room temperature for 1 h. Finally, the membrane was washed 3 times with TBST buffer for 10 min each time and photographed using ECL reagent (Shanghai Qihai Futai Biotechnology Co., Ltd.) for development. Gray value analysis was performed using IPP software. As needed, we set up the TLR4 inhibitor TAK-242 pretreatment group for 6 h, the PI3K inhibitor LY294002 pretreatment group for 12 h and NF-κB p65 inhibitor JSH-23 pretreatment group for 12 h to observe the effect of each inhibitor on macrophage polarization.

### 2.7 Statistical analysis

Statistical analysis was performed using SPSS 19.0 and GraphPad Prism 9.0 software was used for plotting. Measure ment data are expressed as mean ± standard deviation ( $\bar{x}$ ± SD, n = 3) using the independent sample t-test. One-way ANOVA was used for multiple groups, and the LSD-t test was used for further pairwise comparisons. *P*<0.05 was considered statistically significant.

## 3 Results

### 3.1 Effect of *T.cp*-MIF on phenotypic molecules and cytokines associated with the polarization of human THP-1-derived macrophages

**Figure 1:**
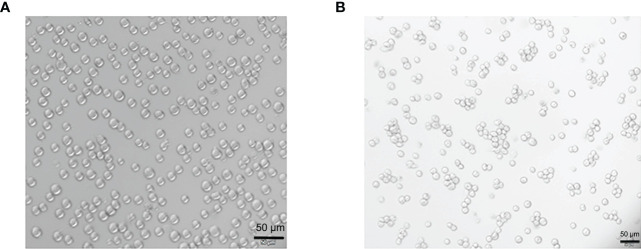
Morphological changes of THP-1 cells treated by Propylene glycol monomethyl ether acetate (PMA). **(A)** The suspension culture cells are round in shape, uniform in size, and they are growing in suspension. **(B)** PMA-induced post-adhesive cells are round or oval in shape, and they are growing anchorage-dependent. Scale bar: 50 µm.

THP-1 cells were round in shape, with uniform cell size and suspended growth; they were round and adherent after 48 h of Propylene glycol monomethyl ether acetate (PMA) treatment (see **Figure 1**). 

**Figure 2:**
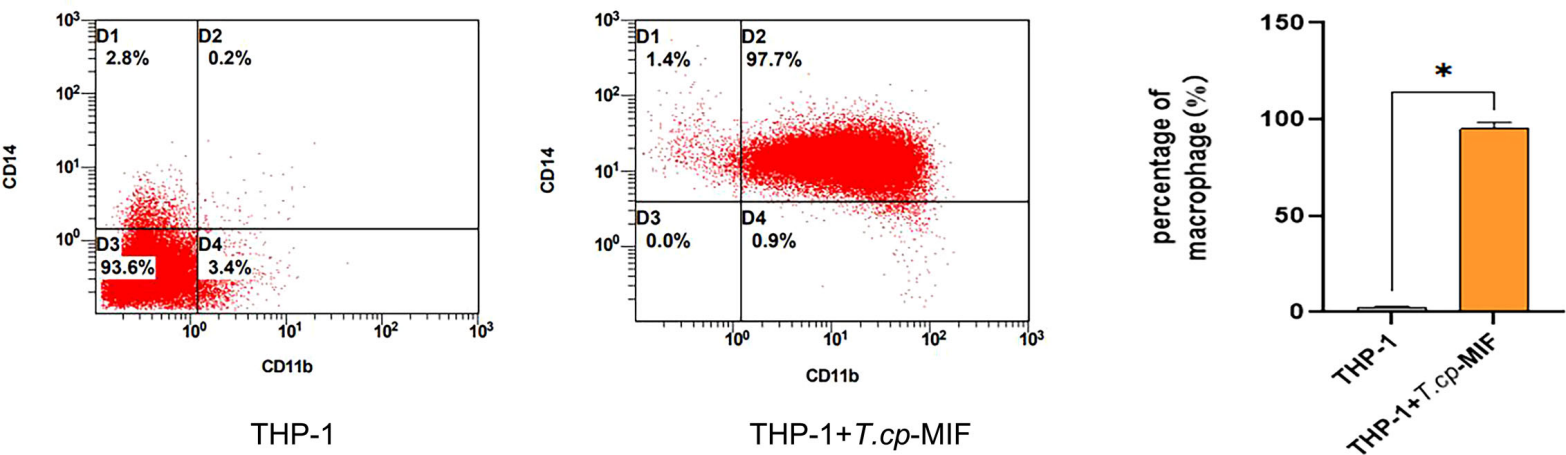
Phenotypic molecular expression in macrophages. The double-positive rate of CD11b and CD14 changed from 0.2% in suspension cells to 97.7% in adnexal cells, and the double-positive rate was greater than 95% for human THP-1-derived macrophages. * indicated *P*<0.05.

**Figure 3:**
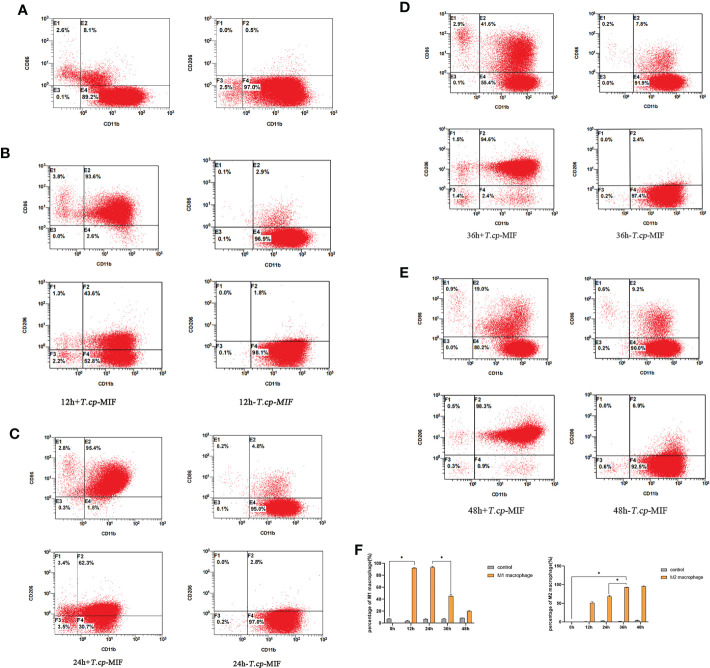
3 The expression of phenotype molecules in M1 and M2 macrophage at different time. **(A–E)** Cell flow plots. **(A)** Double positive rate of CD11b, CD86, CD11b and CD206 in M0 type macrophages after PMA induction. **(B)** The double positive rate of CD11b and CD86 after 12 h of *T.cp*-MIF induction was 93.6% indicating that M0 changed to M1 type macrophages under *T.cp*-MIF induction. **(C)** The double positive rate of CD11b and CD86 was 95.4% in 24 h+*T.cp*-MIF group, indicating that the macrophage was still M1 type at 24 h. **(D)** The double positive rate of CD11b and CD206 after 36 h of *T.cp*-MIF induction was 94.6% indicating that M1 changed to M2 type macrophages under *T.cp*-MIF induction. **(E)** The double positive rate of CD11b and CD206 was 98.3% in 48 h+*T.cp*-MIF group, indicating that the macrophage was still M2 type at 48 h. **(F)** Results are showed as the mean of three times of independent experiments (*x̄ * ± SD, n=3) * indicated *P*<0.05.

The results of flow cytometry showed that CD11b and CD14 double-positive cells accounted for 97.7% (see **Figure 2**), suggesting that THP-1 cells were successfully induced into macrophages, which could be used as the starting point for subsequent M1/M2 polarization for 0 h. The phenotypic molecules of THP-1-derived macrophages, namely M0 type cells, are 8.1% double positive for CD11b and CD86 (M1 type macrophages), and 0.5% for CD11b and CD206 double-positive cells (M2 type macrophages) (see **Figure 3A**). THP-1-derived macrophages were induced by 50 ng/mL *T.cp*-MIF for 12 h and 24 h, and the percentages of CD11b and CD86 double-positive cells were 93.6% and 95.4%, respectively (see **Figures 3B, C, F**). The percentages of CD11b and CD206 double-positive cells were 94.6% and 98.3% after 36 h and 48 h of incubation, respectively (see **Figures 3D–F**).

**Figure 4:**
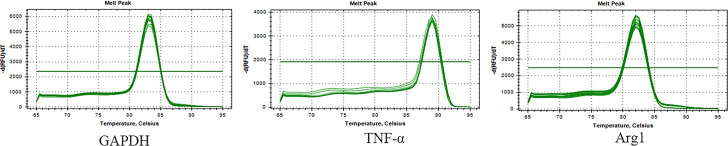
Melting curves of macrophage genes and GAPDH genes. Single peak of the curve indicates the specificity of the designed primer.

**Figure 5:**
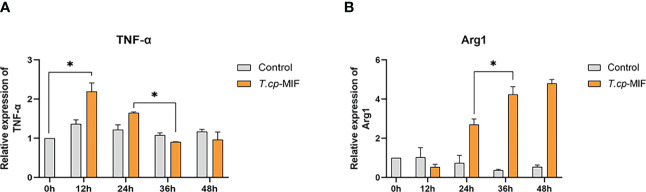
Effects of *T.cp*-MIF on TNF- α and Arg1 at different time. TNF- α and Arg-1 mRNA levels were analyzed by RT-qPCR and normalized against GADPH mRNA. **(A)** the expression of TNF-α. **(B)** the expression of Arg1. * indicated P<0.05. Results are expressed as the mean of three times of independent experiments (*x̄ * ±SD, n=3).

According to the gene melting curve results, the melting curves of TNF-α, Arg1, and GAPDH are single peaks (see **Figure 4**), which indicates the specificity of the designed primers. As shown in **Figure 5**, the mRNA expression of TNF-α gradually increased after induction of *T.cp*-MIF on THP-1 macrophages compared with the control group, reaching a peak at 12 h and being gradually downregulated after 36 h of action (*P*<0.05). Compared with the experimental group at 24 h, the expression of Arg1 mRNA increased significantly after 36 h of action (*P*<0.05). There was no significant difference in the expression of TNF-α and Arg1 mRNA between the control groups (*P*>0.05).

Flow cytometry and real-time fluorescence quantification results showed that macrophages were mainly M1 type at 12 h and 24 h after the action of *T.cp*-MIF on macrophages, and M2 type at 36 h and 48 h after induction.

### 3.2 Transcriptome sequencing analysis of human THP-1 macrophages induced by *T.cp*-MIF

**Figure 6:**
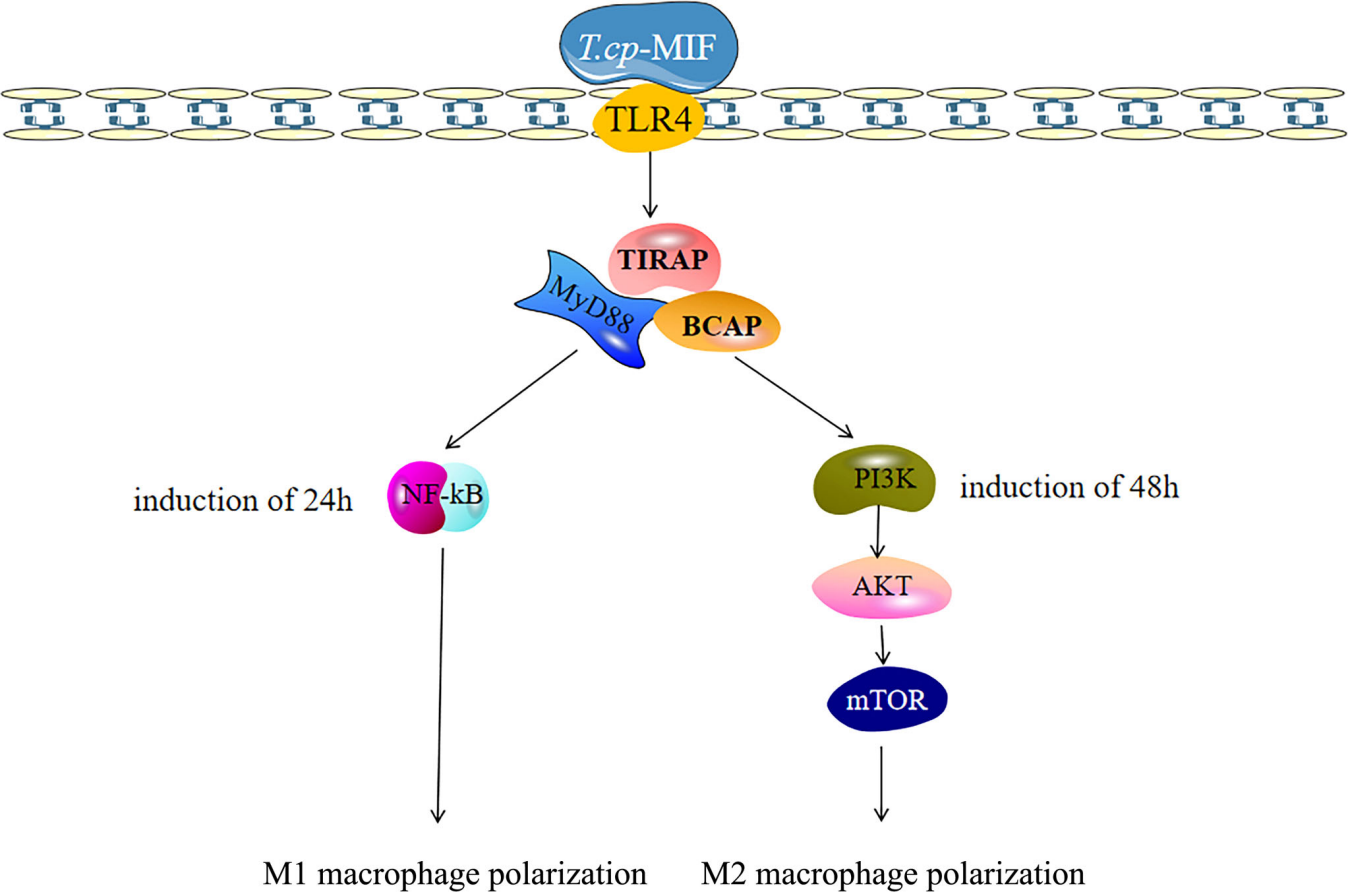
Polarization-related signaling pathway of macrophages was activated after *T.cp*-MIF treatment. The macrophages binded with TLR4, and *T.cp*-MIF activated NF-κB signaling pathway 24 hours after induction. At this time, macrophages showed M1 type polarization, while PI3K/AKT/mTOR signaling pathway is activated at 48 hours, making macrophages show M2 type polarization.

By transcriptome data analysis, the 24 h up-regulated genes in the experimental group after *T.cp*-MIF action mainly included *TLR7*, *TLR4*, etc.; the down-regulated genes were *STPG4*, *MSTN*, etc. The up-regulated genes in the experimental group at 48 h mainly included *SCRG1*, *STPG4*, *TLR7*, etc.; the down-regulated genes included *FSTL5*, *SULT1A4*, etc. The results of the GO functional enrichment analysis of differentially expressed genes are shown in **Tables 4**, **5**. The GO pathways enriched in the 24 h differentially expressed genes were mainly involved in the positive regulation of inflammatory response. The GO pathway enrichment of differentially expressed genes at 48 h included the positive regulation of the inflammatory response and the activity of transmembrane signaling receptors. The results of the KEGG pathway analysis are shown in **Tables 6**, **7**. The 24 h up-regulated pathways in the experimental group included TLRs, NF-κB, chemokines, and other signaling pathways. The main genes enriched on their pathways included *TLR4*, *TLR5*, *TLR7*, and *TLR8*. The main signaling pathways up-regulated in the experimental group at 48 h were mTOR, PI3K-Akt, PPAR and others. Combined with the previous experimental results that the yeast two-hybrid *T.cp*-MIF can bind to TLR4, it is hypothesized that the signaling pathways causing macrophage polarization 24 h and 48 h after the action of *T.cp*-MIF on THP-1-derived macrophages are those shown in **Figure 6**.

**Table 4:** GO term list of significantly differentially expressed genes between the experimental group at 24 h and the control group.

Category	Term	P value
Biological Process	negative regulation of T-helper 1 type immune response	0
	cellular response to interferon-alpha	1.43E-07
	positive regulation of inflammatory response	1.75E-06
Cellular Component	proteinaceous extracellular matrix	1.46E-10
	extracellular space	9.50E-09
	integral component of plasma membrane	9.43E-07
Molecular Function	transcription factor activity, RNA polymerase II proximal promoter sequence-specific DNA binding	0.000148402
	Notch binding	0.001113597
	GABA-A receptor activity	0.000148402

**Table 5:** GO term list of significantly differentially expressed genes between the experimental group at 48 h and 24 h.

Category	ID	P value
Biological Process	regulation of Rho protein signal transduction	1.80E-05
	positive regulation of inflammatory response	2.97E-05
	regulation of cholesterol biosynthetic process	9.70E-05
Cellular Component	extracellular space	2.11E-05
	receptor complex	0.004609
	integral component of plasma membrane	0.007478
Molecular Function	transmembrane signaling receptor activity	0.004398
	G-protein coupled receptor activity	0.009686
	protein binding, bridging	0.000106

**Table 6:** List of KEGG pathways up-regulated and down-regulated between the experimental group at 24 h and the control group 0h.

Up-regulated signaling pathways	Down-regulated signaling pathways
Toll-like receptor signaling pathway	MicroRNAs in cancer
Cytokine-cytokine receptor interaction	cAMP signaling pathway
TNF signaling pathway	Human papillomavirus infection
Cell adhesion molecules (CAMs)	Breast cancer
NF-kappa B signaling pathway	Gastric cancer
Rheumatoid arthritis	Complement and coagulation cascades
Salmonella infection	Glycine, serine, and threonine metabolism

**Table 7:** List of KEGG pathways up-regulated and down-regulated between the experimental group at 48 h and 24 h.

Up-regulated signaling pathways	Down-regulated signaling pathways
mTOR signaling pathway	Cytokine-cytokine receptor interaction
PPAR signaling pathway	Salmonella infection
PI3K-Akt signaling pathway	Cytosolic DNA-sensing pathway
AMPK signaling pathway	Chemokine signaling pathway

### 3.3 Western blot assay to detect the expression levels of TLR4, NF-κBp65, PI3K, and Akt protein and protein phosphorylation

**Figure 7:**
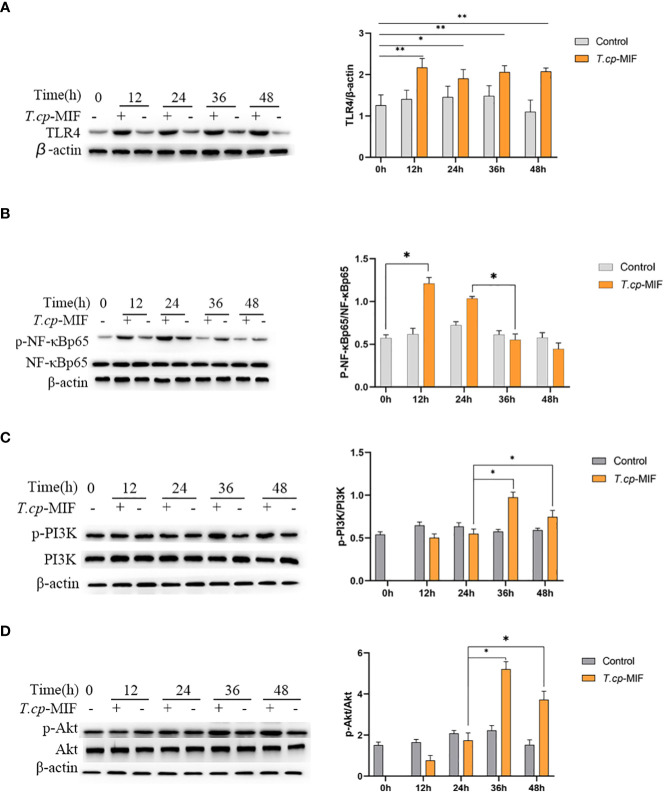
Effect of *T.cp*-MIF on the expression of macrophage polarization-related proteins in different time. **(A–D)** The protein expression levers of TLR4, NF-κBp65, PI3K and AKT were analyzed by western blot and normalized against β-actin expression. * indicated *P*<0.05. Results are showed as the mean of three times of independent experiments (*x̄ *±SD, n=3).

Based on the transcriptome sequencing results of *T.cp*-MIF-induced THP-1-derived macrophages for 24 h and 48 h, we can infer that TLR4, NF-κB, PI3K/Akt, and other pathways may be involved in the process of macrophage polarization. Therefore, a Western blot was performed to detect the expression level of these proteins and signaling pathways in the polarization. The results are shown in **Figure 7A**. TLR4 protein expression was significantly increased in each experimental group after *T.cp*-MIF induction compared with the control group (*P*<0.05), and there was no significant difference between experimental groups (*P*>0.05). Compared with the control, the expression of p-NF-κBp65 protein was significantly up-regulated at 12 h and 24 h after the effect of *T.cp*-MIF, and significantly down-regulated at 36 h and 48 h after induction (*P*<0.05) (see **Figure 7B**). Compared with the control, the expressions of p-PI3K and p-Akt proteins were not significantly different at 12 h and 24 h after the effect of *T.cp*-MIF (*P*>0.05); while the phosphorylation levels of PI3K and Akt proteins were significantly increased at 36 h and 48 h after induction (*P*<0.05) (see **Figures 7C, D**). There was no statistical difference in the expression levels of TLR4, p-NF-κBp65, p-PI3K, and p-Akt proteins in each control group (*P*>0.05).

### 3.4 Effects of TLR4 inhibitor TAK-242 on macrophage polarization and the expression levels of TLR4, NF-κBp65, PI3K, and Akt protein and protein phosphorylation

**Figure 8:**
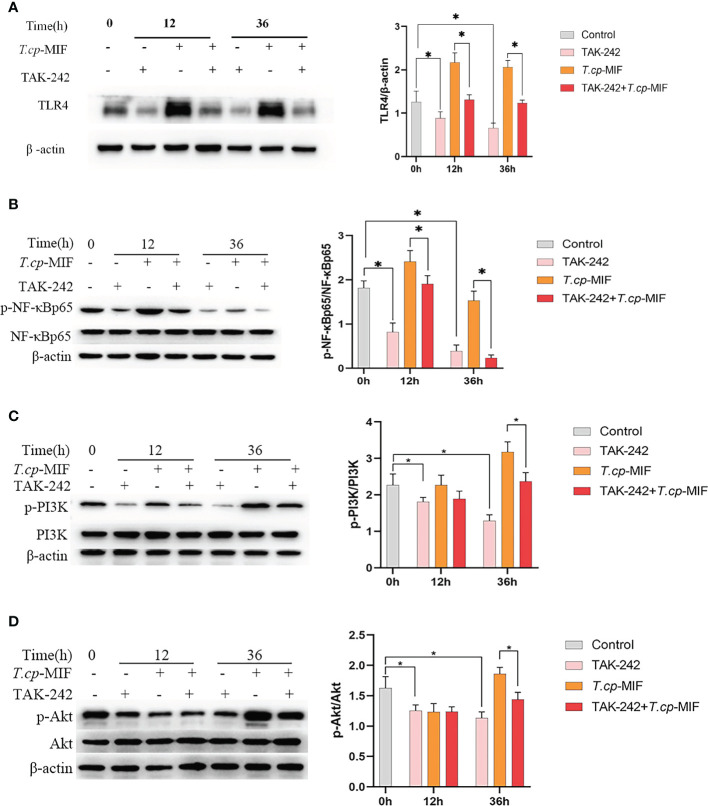
The effect of TAK-242 on the expression of macrophage polarization-related proteins. **(A–D)** The cells were pretreated with TAK-242 (the TLR4 inhibitor) for 6 h, and the expression levels of TLR4, NF-κBp65, p-NF-κBp65, PI3K, p-PI3K, Akt and p-Akt were detected by western blot. * indicated *P*<0.05. Results are showed as the mean of three times of independent experiments (*x̄ *±SD, n=3).

**Figure 9:**
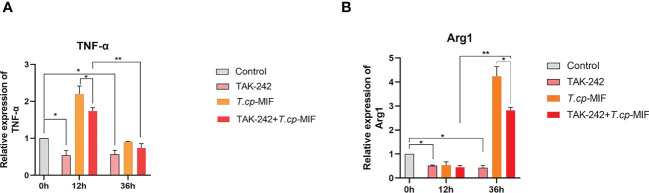
The effect of TAK-242 on the expression of TNF-α and Arg1. **(A)** the expression of TNF-α. **(B)** the expression of Arg1. * indicated *P*<0.05, ** indicated *P*<0.01. Results are showed as the mean of three times of independent experiments (*x̄ *±SD, n=3).

Based on the above experimental results, to further verify the role of TLR4/NF-κB and PI3K/Akt pathways in *T.cp*-MIF-induced macrophage polarization, macrophages were pretreated with TLR4 inhibitor TAK-242 and induced with *T.cp* -MIF for 12 h (M1 type) and 36 h (M2 type). The changes in TLR4, NF-κBp65, p-NF-κBp65, PI3K, p-PI3K, Akt, and p-Akt protein expression levels in each group of cells were detected by Western blot. The results are shown in **Figures 8A, B**. Compared with the *T.cp*-MIF group, the *T.cp*-MIF+TAK-242 group at 12 h and 36 h showed a significantly inhibited expressions of TLR4 and p-NF-κBp65 proteins (*P*<0.05). As shown in **Figures 8C, D**, compared with the *T.cp*-MIF group, the TLR4 receptor inhibitor TAK-242 can inhibit the expression of p-PI3K and p-Akt proteins for 36 h (*P*<0.05); while the expressions of p-PI3K and p-Akt proteins in the *T.cp*-MIF+TAK-242 group for 12 h compared with those in the *T.cp*-MIF group were not statistically different (*P*>0.05). Real-time fluorescence quantitative PCR was performed to detect the expression of TNF-α and Arg1 mRNA in each group of cells. The results are shown in **Figure 9**. Compared with the control group, the expressions of TNF-α and Arg1 in the 12 h and 36 h TAK-242 groups were lower than those in the control group (*P*<0.05). Compared with the *T.cp*-MIF group, the expression of TNF-α in the *T.cp*-MIF+TAK-242 group at 12 h was significantly decreased (*P*<0.05), and the expression of Arg1 in the *T.cp*-MIF+TAK-242 group at 36 h was significantly decreased (*P*<0.05). TNF-α expression was significantly decreased (*P*<0.01) and Arg1 expression was significantly increased (*P*<0.01) in the 36 h *T.cp*-MIF+TAK-242 group compared with their values in the 12 h *T.cp*-MIF+TAK-242 group.

### 3.5 Effects of PI3K inhibitor LY294002 on macrophage polarization and the levels of NF-κBp65, PI3K, and Akt protein and protein phosphorylation

**Figure 10:**
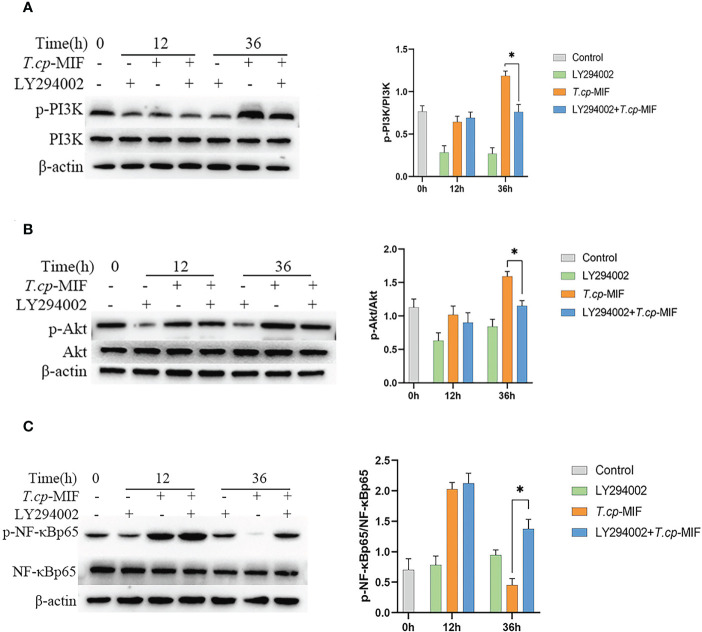
The effect of LY294002 on the expression of macrophage polarization-related proteins. **(A–C)** The cells were pretreated with LY294002 (the PI3K inhibitor) for 12 h, and the expression levels of PI3K, p-PI3K, Akt, p-Akt, NF-κBp65 and p-NF-κBp65 were detected by western blot. * indicated *P*<0.05. Results are showed as the mean of three times of independent experiments (*x̄ *±SD, n=3).

**Figure 11:**
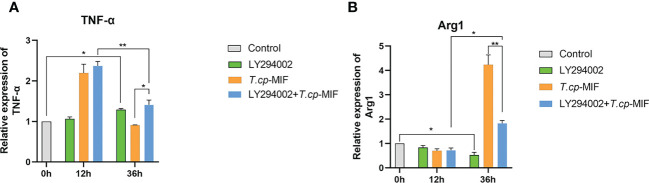
The effect of LY294002 on the expression of TNF-α and Arg1. **(A)** the expression of TNF-α. **(B)** the expression of Arg1. * indicated *P*<0.05, ** indicated *P*<0.01. Results are showed as the mean of three times of independent experiments (*x̄ *±SD, n=3).

After pretreatment with the PI3K inhibitor LY294002 with *T.cp*-MIF to induce cells for 12 h and 36 h, the expression levels of TNF-α and Arg1 mRNA in macrophages were detected by real-time fluorescence quantitative PCR, and Western blot was performed to detect the changes in NF-κBp65, p-NF-κBp65, PI3K, p-PI3K, Akt, and p-Akt protein levels. The results are shown in **Figures 10A–C**. Compared with the *T.cp*-MIF group, the expression levels of p-PI3K, p-Akt and p-NF-κBp65 in the *T.cp*-MIF+ LY294002 group at 12 h were not statistically different (*P*>0.05); the protein expression of p-PI3K and p-Akt at 36 h was significantly down-regulated, and the p-NF-κBp65 protein expression levels were significantly increased (*P*<0.05). We used real-time fluorescence quantitative PCR to detect the expression of TNF-α and Arg1 mRNA in each group of cells, and the results are shown in **Figure 11**. Compared with the control group, the expression of TNF-α was significantly increased (*P*<0.05) and that of Arg1 was significantly decreased (*P*<0.05) in the 36 h inhibitor group. Compared with that in the *T.cp-*MIF group, TNF-α expression was significantly increased (*P*<0.05) and Arg1 expression was significantly decreased (*P*<0.01) in the 36 h *T.cp*-MIF+LY294002 group. Compared with the 12 h *T.cp*-MIF+ LY294002 group, the 36 h *T.cp-*MIF+ LY294002 group showed a significant decrease in TNF-α (*P*<0.01) and a significant increase in Arg1 expression (*P*<0.05).

### 3.6 Effects of the NF-κBp65 inhibitor JSH-23 on macrophage polarization and NF-κBp65 expression levels

**Figure 12:**
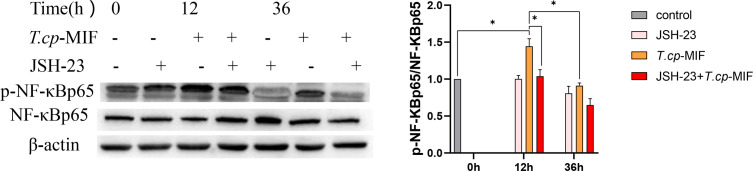
The effect of JSH-23 on the expression of macrophage polarization-related proteins. The cells were pretreated with JSH-23 (the NF-κB inhibitor) for 12 h, and the expression levels of NF-κBp65 and p-NF-κBp65 were detected by western blot. * indicated *P*<0.05. Results are showed as the mean of three times of independent experiments (*x̄ *±SD, n=3).

After pretreatment with the NF-κBp65 inhibitor JSH-23 with *T.cp*-MIF to induce cells for 12 h, a Western blot was performed to detect changes in NF-κBp65 and p-NF-κBp65 protein levels. The results are shown in **Figure 12**. Compared with the control group, the expression levels of p-NF-κBp65 in *T.cp*-MIF group were significantly increased (*P*<0.05). Compared with the *T.cp*-MIF group, the expression levels of p-NF-κBp65 in the *T.cp*-MIF+ JSH-23 group at 12 h were significantly down-regulated (*P*<0.05); the protein expression levels of p-NF-κBp65 in *T.cp*-MIF+ JSH-23 group at 36 h were significantly down-regulated (*P*<0.05).

## 4 Discussion

*Thelazia callipaeda*, as ocular parasites, can secrete a parasite-derived MIF (*T.cp*-MIF) that is highly similar to human-derived MIF in structure and function and can act on macrophages to cause M1/M2 type polarization, whose role in the immune interaction mechanism between conjunctival sucking nematodes and hosts is of interest. When many parasites are infected, macrophages will show the dominant activation of the M2 type or show the dynamic polarization of the M1 to the M2 type, which plays an important role in parasite immune evasion by suppressing the inflammatory response, promoting chronic infection, and favoring long-term parasitism (15–17). THP-1 cells are widely used in the study of monocyte and macrophage-related mechanisms and signaling pathways. They have more morphological and functional characteristics similar to human primary monocytes (including cell differentiation markers), relative to human peripheral blood mononuclear cells (PBMC). They are also more easily cultured and expanded in the laboratory and have a more stable genetic background, without the problem of the individual variability of PBMC (18). In the present study, we detected the phenotypic molecules CD11b, CD86, and CD206, as well as the mRNA expression levels of TNF-α and Arg1. We confirmed that 12 h after the induction of worm-derived *T.cp*-MIF, macrophages began to exhibit M1-type polarization (93.6% of CD11b and CD86 double-positive cells and a significant increase in mRNA expression of TNF-α). Additionally, 36 h after the induction of worm-derived *T.cp*-MIF, macrophages started to show M2-type polarization (94.6% of CD11b and CD206 double-positive cells and a significant increase in the mRNA expression of Arg1). This process manifests itself as a dynamic polarization from M1 to M2 type.

It is known that macrophage polarization is a complex process with multifactorial interactions and is regulated by multiple intracellular and extracellular signaling molecules (19, 20). Studies have shown that a variety of TLR ligands bind to TLRs, activate TLRs and other related macrophage polarization signaling pathways, and produce pro-inflammatory or anti-inflammatory factors, which in turn affects macrophage polarization. In this study, it was found that the expression of TLR4 protein was significantly increased in each experimental group after the action of *T.cp*-MIF on THP-1-derived macrophages. The expression of TLR4, p-NF-κBp65, p-PI3K, and p-Akt proteins was significantly decreased when THP-1-derived macrophages were treated with TLR4 inhibitor (*P*<0.05), which verified the correlation between TLR4 and its downstream NF-κBp65, PI3K, and Akt proteins. By further comparing the protein expression and cytokine expression in the TAK-242 and TAK-242+*T.cp*-MIF groups, it can be preliminarily confirmed that the mechanism of dynamic polarization caused by *T.cp*-MIF acting on THP-1-derived macrophages may be the binding to TLR4 and then activating the downstream macrophage M1/M2 type polarization-related pathway.

The polarization of macrophages is not only affected by signaling molecules and chemokines, but also tightly and precisely regulated by receptor stimulation or signaling cascades triggered by intracellular regulatory proteins. The main pathways affecting macrophage polarization include NF-κB, JAK/STAT, PI3K/Akt, and mTOR (21–25). TLRs can regulate the expression of inflammatory factors such as IL-12 and TNF-α by activating NF-κB and AP-1 transcriptional regulators. At the same time, TLRs, as transmembrane proteins, can affect macrophage polarization by mediating JNK and PI3K/Akt pathways (26). Activation of the PI3K/Akt pathway has inhibitory effects on TLR-mediated macrophage inflammatory responses, while the PI3K signaling pathway can inhibit M1-type polarization and promote M2 type polarization by inhibiting the NF-κB pathway (27). Based on the experimental results, it is hypothesized that *T.cp*-MIF induced the M1 type polarization of macrophages through the TLR4/NF-κB pathway after 12 h of action by binding to TLR4. After 36 h, it activates the PI3K/Akt pathway, inhibits the phosphorylation of NF-κB protein, and induces M2 type polarization.

According to the differential transcriptomic expression analysis, the expression of TLR7 was also significantly increased. As one of the members of the TLRs family, its binding to ligands induced the expression of IFN-γ and IL-6 by activating the transcription and translation-related proteins NF-κB and MAPK-ERK1/2 through a MyD88-dependent pathway (28, 29). It was found that TLR7 is also involved in the dynamic polarization process of macrophages (30, 31). Therefore, the results of this study suggest the need for further understanding of the role of TLR7 in immunity to *Thelazia callipaeda* infection, particularly in macrophage polarization.

The phenotype and function of macrophages are heterogeneous and plastic, and under different stimuli, macrophages can polarize into functionally distinct M1 and M2 types, which play different roles in the host (32). Among the M2 type macrophage polarization, four subtypes are classified according to receptor expression and function: M2a, M2b, M2c, and M2d (33, 34). The M2a type macrophages can be induced by IL-4 and IL-13, specifically express CD206 and CD301, and exert anti-inflammatory activity and tissue remodeling effects (35, 36). The M2b type is induced by IL-1, LPS, and immune complexes (37), which express CD64, TNF-α, and other cytokines (38) to regulate the immune response. The M2c type is induced by IL-10 and glucocorticoids and expressesd TGF-β and IL-10, involved in tissue damage repair and matrix reconstruction (39, 40). The M2d macrophages are induced by TLR agonists through adenosine receptors and highly express IL-10, which can induce angiogenesis and promote tumor cell growth (41). However, it needs to be further clarified which subtype of M2 type polarization is induced by parasite-derived *T.cp*-MIF, and how it plays the role of immune regulation and immune evasion in *T. callipaeda* infection also deserves further investigation.

In the present study, the molecular mechanism of *T.cp*-MIF-induced polarization of human macrophages was only investigated by TLR4, NF-κB, PI3K, and Akt pathways based on transcriptome screening data. However, the signaling pathways of macrophage polarization are intricate and complex, and the signaling between TLR4/NF-κB and PI3K/Akt pathways have not been elucidated. In addition, the roles and network regulatory relationships of TLR7 and other highly differentially expressed genes in macrophage polarization-related signaling pathways also deserve further study. Therefore, more in-depth studies based on transcriptome data are needed to further investigate parasit-derived *T.cp*-MIF-induced macrophage polarization and the mutual regulatory relationships between the pathways, as well as to gain insight into how insect-derived *T.cp*-MIF regulates *T. callipaeda* interactions with the host.

## 5 Conclusions

The mechanism of *T.cp*-MIF-induced dynamic polarization of human THP-1-derived macrophages from the M1 to M2 type is related to TLR4 binding. It can affect M1 macrophage type polarization by activating its downstream NF-κB pathway first, further activating the PI3K/Akt pathway, and inhibiting NF-κB phosphorylation to promote M2 type macrophage polarization.

## Data availability statement

The datasets presented in this study can be found in online repositories. The names of the repository/repositories and accession number(s) can be found below: PRJNA902947 (BioProject).

## Author contributions

All authors listed have made a substantial, direct, and intellectual contribution to the work, and approved it for publication.
